# UBE3A and Its Link With Autism

**DOI:** 10.3389/fnmol.2018.00448

**Published:** 2018-12-04

**Authors:** Naman Vatsa, Nihar Ranjan Jana

**Affiliations:** ^1^Cellular and Molecular Neuroscience Laboratory, National Brain Research Centre, Gurugram, India; ^2^School of Bioscience, Indian Institute of Technology, Kharagpur, India

**Keywords:** UBE3A, autism, Angelman syndrome, synaptic plasticity, animal models

## Abstract

UBE3A is a dual function protein consisting of ubiquitin ligase as well as transcriptional co-activator function. *UBE3A* gene is imprinted in the brain with preferential maternal-specific expression particularly in the neuron and loss of activity of the maternally inherited *UBE3A* causes Angelman syndrome (AS), characterized by severe mental retardation, lack of speech, seizures and autistic features. Interestingly, duplication, triplication, or gain-of-function mutations in the *UBE3A* gene are also linked with autism clinically distinguished by social impairments and stereotyped behaviors. These findings indicate that the expression and activity of UBE3A must be tightly regulated during brain development and UBE3A might be playing a crucial role in controlling synaptic function and plasticity through proteasome-mediated degradation as well as transcriptional regulation of its target proteins. In fact, several recent reports demonstrated the role of UBE3A in the modulation of synaptic function and plasticity. This review focuses on the critical role of UBE3A in regulating the synaptic function and how its altered activity is associated with autism.

## Introduction

International classification of diseases (ICD-10) describes autism as an early onset neurodevelopmental disorder with three major characteristics feature: lack of communication, lack of social skills, and repetitive stereotyped behavior. With the extensive study in multiple new cases, the Diagnostic and Statistical Manual of Mental Disorders (DSM-V) updated this definition in 2013, wherein, delayed language development was removed from the list of criteria and autism covered a host of diseases redefining itself as an autism spectrum disorder (ASD). First reports of autism were made by Leo Kanner and Hans Asperger in 1943–44. Both Kanner and Asperger defined a set of children withdrawn in their own world and failing to express themselves in social settings. Kanner termed this condition *infantile autism* while Asperger called it *autistic psychopathy*, both deriving their descriptions from schizophrenia and psychosis (Kanner, 1968; Asperger, 1991).

ASD has come a long way from its mid-twenties view of childhood psychosis to today, where it can be categorized according to its developmental pattern and co-morbidity with other diseases. ASD is a category of developmental disorders each with different behavioral phenotypes and severity and autism is the most common ASD. Improved diagnosis along with increased understanding of environmental risk factors has led to a considerable rise in the number of report worldwide. WHO report states that on an average 1 in 160 children's are suffering from ASD around the world, and it may occur more in males than in females. While the number of autistic cases continues to increase, the real cause behind the disease still remains a mystery. Reports suggest that environmental factors such as *in utero* exposure to alcohol, recreational drugs, pesticides, prenatal valproate exposure, nutritional deficiency, and maternal infections may hamper neural development leading to ASD (Roberts et al., 2007; Christensen et al., 2013; Volk et al., 2013; Karimi et al., 2017). A growing body of research also points at the complicated genetic etiology of ASD with almost 800 genes enlisted in the autism database (AUTdb). Synaptic scaffold (*SHANK3*) (Berkel et al., 2010), calcium ion channel (*CACNA1C*) that are known to be associated with Timothy syndrome (de la Torre-Ubieta et al., 2016) and GABA receptor genes (*GABRB3, GABRA5, GABRG3*) (Kim et al., 2006; Klauck, 2006; Vorstman et al., 2006), genes encoding neuronal surface proteins (*NLGN4X*), intracellular trans-membrane proteins of the Golgi complex (*POMGNT1*), and genes responsible for proper cerebellar function (*SYNE1*) are important few names among multitude of genetic factors that may cause ASD (Vorstman et al., 2017). Maternally inherited overexpression of *UBE3A* is currently thought to be the main cause underlying the Dup15q syndrome having typical autistic phenotypes (Cook et al., 1997; Urraca et al., 2013). Moreover, the loss of function of maternally inherited *UBE3A* is also associated with Angelman syndrome (AS), a neurodevelopmental disorder with severe mental retardation along with autistic features (Malzac et al., 1998; Moncla et al., 1999), thus making *UBE3A* a potential candidate gene to be studied for better understanding of autism. This review primarily focuses on the regulation of synaptic function and plasticity by UBE3A and how its abnormal function is associated with autism.

## The 15q11-q13 Region: A Link Between *UBE3A* and Autism

The *UBE3A* gene is located on the proximal arm of the 15th chromosome at the q11–q13 site in humans, while it is found on the 7th chromosome in mouse (Kishino and Wagstaff, 1998). Interestingly, majority of AS cases are due to the deletion of maternally derived copy of the 15q11–q13 chromosomal region. This region comprises many genes, one of which is *UBE3A* (Saitoh et al., 1997). Furthermore, discovery of a various point mutations in the *UBE3A* gene in 5–10% cases of AS strongly implicated it as a causative factor for AS (Malzac et al., 1998; Lossie et al., 2001). AS is characterized by severe mental retardation, developmental delay, cognitive impairment, seizures, and excessive laughter a feature that led Dr. Harry Angelman name this disease the *happy puppet syndrome*. Other than being sociable, children with AS exhibit many features overlapping with autism. This has resulted in a debate among researchers, where some consider AS and ASD comorbid (Steffenburg et al., 1996), while others believe AS to be distinct disorder (Williams et al., 2001). Autism phenotypes also has been described to be associated with a number of other neuro-genetic disorders such as Rett syndrome, Tuberous sclerosis, and Fragile X syndrome (FXS) (Gillberg, 1998). Recent studies have indicated that in about 20% cases of autism, a high number of small genomic DNA copy number variations (CNVs) were present (Sebat et al., 2007; Morrow et al., 2008; Weiss et al., 2008; Glessner et al., 2009). Interestingly, previous studies have also shown that maternally inherited 15q11–13 duplications and triplications are among the most common genomic CNVs identified in patients with autism (Cook et al., 1997; Schroer et al., 1998; Thomas et al., 2003). Individuals having an inverted duplication of an extra maternal 15q11–13 segment (dup15), show mild autistic features, whereas individuals with two extra copies (triplication) resulting from an isodicentric extranumerary chromosome (idic15) display most of the autistic symptom whereas the paternal duplication of this region does not lead to autism (Hogart et al., 2010). These studies suggest the involvement of imprinted genes lying within the duplicated chromosomal region as the cause of autism in these patients.

Interestingly, *UBE3A*, the AS gene, was identified as one of genes within the 15q11–q13 region which was solely expressed from the maternal allele in mature neurons (Albrecht et al., 1997). Further, linkage disequilibrium at the 5′ end of *UBE3A* in families of autistic children (Nurmi et al., 2001; Shao et al., 2003) and paternally derived mutations at the same site not resulting in an autistic phenotype makes *UBE3A* a candidate gene for susceptibility to autism and related disorders. Earlier studies had suggested that *ATP10A*, a gene also lying at the 15q11–q13 site and expressed exclusively from the maternal chromosome (Meguro et al., 2001; Kashiwagi et al., 2003) could be another candidate gene for autism. However, this theory was later refuted, as *ATP10A* was not found to be maternally imprinted (Kayashima et al., 2003; Nakatani et al., 2009). Other genes coding for GABA_A_ (γ-aminobutyric acid type A) receptor subunits β3, α5, γ3, and CYFIPI (cytoplasmic FMRP-interacting protein 1) are also reported in autism (Nishimura et al., 2007; Hogart et al., 2010), which suggests the involvement of genes other than *UBE3A* lying within the 15q11–q13 region in autistic pathophysiology.

## UBE3A: The Jack of All Trades

**Figure 1:**
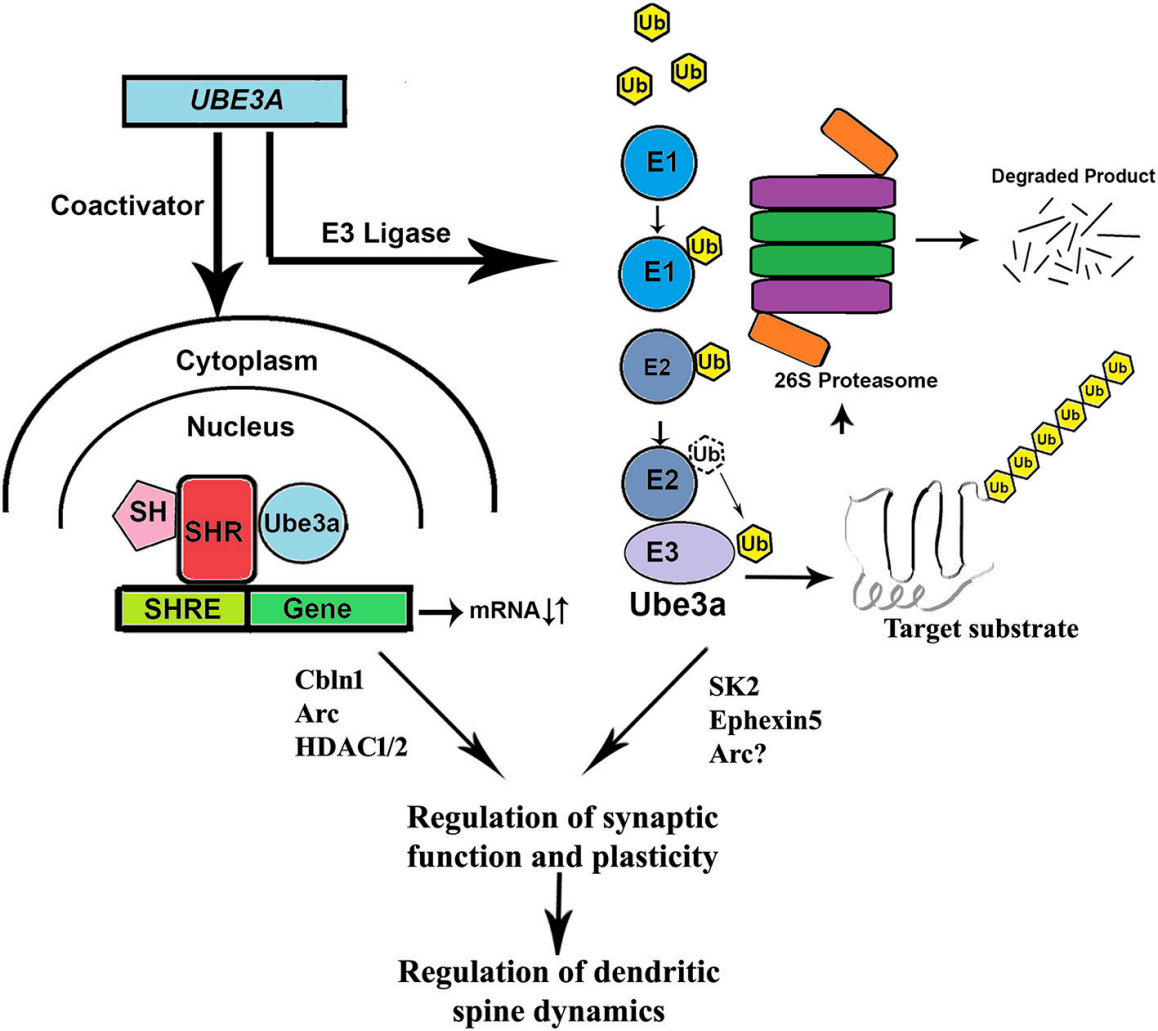
Role of UBE3A in regulating synaptic function and plasticity. Being an ubiquitin ligase, UBE3A could selectively target various synaptic proteins (Ephexin5, SK2 Arc etc.) through proteasome-mediated degradation and thereby regulates synaptic function and plasticity. UBE3A also could regulate the expression of various genes (Cbln1, Arc, HDAC1, and 2 etc.) linked with synaptic function by the virtue of its coactivator function. Loss of function or overactivation of UBE3A could potentially altered synaptic function and plasticity, the underlying cause of many behavioral deficits observed in both AS and autism. SH, steroid hormone; SHR, steroid hormone receptor; SHRE, steroid hormone response element; Ub, ubiquitin; E1, ubiquitin activating enzyme; E2, ubiquitin conjugating enzyme; E3, ubiquitin ligase.

The *UBE3A* gene encodes a 100 kDa protein that is functionally characterized as an E3 ubiquitin ligase belonging to the HECT (homologous to E6-AP C-terminus) domain family. As a part of the ubiquitin proteasome system, ubiquitin ligases (such as UBE3A) selectively target specific proteins for their ubiquitination and subsequent degradation by proteasome (Hershko and Ciechanover, 1992). Interestingly, UBE3A was initially discovered in degrading tumor suppressor P53 protein by interacting with the viral E6 oncoprotein in cells infected with human papiloma virus (Scheffner et al., 1993). Apart from its ligase activity, UBE3A also acts as a transcriptional co-activator (Nawaz et al., 1999; Smith et al., 2002; Khan et al., 2006) and found to regulate cell cycle (Oda et al., 1999; Mishra and Jana, 2008; Mishra et al., 2009a), synaptic function and plasticity (Reiter et al., 2006; Greer et al., 2010; Margolis et al., 2010; Kaphzan et al., 2011, 2012; Mabb et al., 2011; Sun et al., 2015), and cellular protein quality control (Mishra et al., 2008, 2009b; Mulherkar et al., 2009). Because of the link of UBE3A with AS and autism, researcher becomes increasingly interested to understand the regulation of synaptic function and plasticity by UBE3A. In fact, UBE3A has been shown to regulate synaptic plasticity and learning and memory formation by targeting activity regulated cytoskeleton-associated protein (Arc), a Rho guanine nucleotide exchange factor (Ephexin5), and small conductance calcium-activated potassium channel (SK2) (Greer et al., 2010; Margolis et al., 2010; Sun et al., 2015). The expression of UBE3A is increased during experience-driven neuronal activity and increased UBE3A up-regulates excitatory synapse formation by regulating the level of Arc, a synaptic protein that induces internalization of AMPA types of glutamate receptor(Greer et al., 2010). However, subsequent study failed to establish Arc as substrate of UBE3A and rather it is transcriptionally regulated by UBE3A (Kuhnle et al., 2013). UBE3A also ubiquitinates SK2 and promotes its endocytosis resulting in increased NMDA receptor activation thus regulating synaptic plasticity (Sun et al., 2015). The *Ube3a*-maternal deficient mice (AS model mice) generated by Jiang et al reproduced many characteristic features of AS including cognitive and motor deficits, audiogenic seizure, anxiety-like behavior, disturbances in circadian clock, and sleep homeostasis (Jiang et al., 1998; Heck et al., 2008; Godavarthi et al., 2012; Shi et al., 2015). These AS mice also shows defect in hippocampal calcium/calmodulin dependent protein kinase-II and long-term potentiation, experience-dependent synaptic plasticity, and imbalance of excitatory/inhibitory circuitry (Weeber et al., 2003; Yashiro et al., 2009; Sato and Stryker, 2010; Wallace et al., 2012). Many of these abnormalities in AS mice could be due to the altered level of SK2, Ephexin5, Arc, or some other novel substrates of UBE3A (shown in Figure 1).

## Overdosage of UBE3A in Autism: Study on Animal Model

The first mouse model of autism was developed by Nakatani et al. (2009), where they used chromosomal engineering to duplicate a 6.3 Mb long mouse region orthologous to the human 15q11–13. This region encompassed many genes like *Ndn, Ube3a, Atp10a, Gabrb3, Gabrb5, Gabrg3*, and *Herc2*. Although this model showed behavioral abnormalities, they were mostly associated with paternally inherited duplication of the region. Since this result was not similar with maternal specific inheritance pattern of 15q11–13 duplications as observed in Dup15q syndrome patients (Cook et al., 1997; Nakatani et al., 2009; Urraca et al., 2013; Ellegood et al., 2015), this model did not truly represent the autistic phenotype. In order to delineate the role of over dosage of *UBE3A* in autism, Smith et al. generated a mouse model over expressing flagged tagged Ube3a by using bacterial artificial chromosome (BAC) recombinant technique. They inserted the 162 kb fragment of mouse chromosome 7 which contained the entire 78 kb coding sequence of *Ube3a* gene as well as its 63 kb 5′ and 21 kb 3′ extragenic sequence into FVB embryos, which were subsequently bred to produce single or double copy transgenic mice (Smith et al., 2011). The flagged tagged *Ube3a* was expressed across all brain regions. In accordance with the idic15 autism phenotype, the mouse with three-fold increase in *Ube3a* (2xTg) had impaired social behavior and communication along with increase in repetitive behavior, which is the hallmark of autism. And similar to the dup15 phenotype, the mouse with two fold increase in brain *Ube3a* (1xTg) had a weaker autism penetrance with few of behavioral deficits. This model suggested that *Ube3a* is a dose sensitive gene and it is required in triplication to reconstitute the full set of symptoms of autism in a mouse model system.

Another mouse model of ASD carrying a point mutation in neuroligin 3 (*NLGN3*) showed increased GABAergic transmission, while mouse model for Rett syndrome (where neurons were depleted of *MeCP2*) showed decreased GABAergic transmission without affecting glutamatergic transmission (Chao et al., 2010). In contrast, the Ube3a overexpression model showed reduction in glutamatergic synaptic transmission with minimal effect on GABAergic transmission in the neuron. There was no difference in the number of synapse and dendritic spine. However, presynaptic release probability and synaptic glutamate concentration was found to be significantly reduced in Ube3a overexpressing mice. This may have resulted in a reduction of mEPSCs, suggesting that excess Ube3a acts at multiple distinct sites within the pre-post synaptic compartment of neuronal circuitry and may impair excitatory synaptic transmission leaving the GABAergic synaptic transmission intact. Similar reports by Yashiro et al. (2009) showing reduced frequency of mEPSCs in visual cortex (layer 2/3) pyramidal neurons and reduction in glutamatergic transmission as shown by Greer et al. (2010) in AS mouse model, links both the loss and gain of function of Ube3a to abnormal neurodevelopment. These findings also elucidate the molecular and circuit mechanisms behind both types of mutations.

However, the link between increased Ube3a expression and the autistic phenotype still remained unclear. To clarify this association, Krishnan et al. (2017) identified 190 up-regulated and 408 down-regulated genes in *Ube3a* (2xTg) mice which were enriched for glutamatergic synaptic transmission. They found *Cbln1*, a gene which binds to Nrxn1/2/3 and postsynaptic GRID1/2 and is dose dependently affected by Ube3a. Both *NRXN1/2/3* and *GRID1/2* genes were found mutated in ASD in a network protein-protein interaction study (Matsuda et al., 2010; Matsuda and Yuzaki, 2011; Wei et al., 2012; Elegheert et al., 2016). They reported down regulation of *Cbln1* in both *Ube3a* (1xTg) and *Ube3a* (2xTg) mouse models. Krishnan et al. also observed social deficits similar to *Ube3a* (2xTg) mouse in animals with *Cbln1* deleted from VGLUT neurons specifically in the ventral tegmental area (VTA). They also reported that recurrent seizures reduced *Cbln1* expression along with impaired sociability. This result was supported by an earlier report that showed down-regulation of Cbln1 mRNA by increased neuronal activity after status epilepticus (Iijima et al., 2009). This study suggested that Ube3a was needed for seizure induced decrease in sociability and the repression of *Cbln1*. Restoring *CBLN1* expression in VGLUT neurons of VTA rescued impaired sociability. Another study has shown increased level and activity of HDAC1/2 in AS mice brain (Jamal et al., 2017). Since HDAC2 negatively regulates synaptic plasticity and memory formation (Guan et al., 2009), its altered activity could lead to synaptic dysfunction in UBE3A-linked AS and autism. These findings indicate how transcriptional deregulation by Ube3a could be associated with abnormal synaptic function (see Figure 1).

In humans, *UBE3A* is expressed at eight alternatively spliced transcripts that encode for three protein isoforms (Yamamoto et al., 1997; LaSalle et al., 2015), whereas in mouse *Ube3a* expresses at least three alternatively spliced transcripts (Yamamoto et al., 1997; Miao et al., 2013). The function of each isoform of Ube3a in the brain is not clearly understood. Mio et al. suggest that isoform 2 of Ube3a is responsible for specification of apical dendrites and dendritic polarization of pyramidal neurons in the mouse cortex (Miao et al., 2013). Ube3a isoform 1 has been reported to regulate dendritic spine development by sequestering miR-134—Limk1 complex, an enzyme involved in synaptogenesis (Valluy et al., 2015). These studies suggest that each isoform of Ube3a is involved in some form of neuronal development, synaptogenesis and plasticity. Thus, mouse models were needed in order to study the effect of over expression of different isoforms with respect to autism. Copping et al. (2017), using the Tetracycline–Off (Tet-Off) expression system, developed and characterized a mouse model for autism, which over expressed Ube3a isoform 2 (analogous to human *UBE3A* isoform 3) in excitatory neurons. Ube3a isoform 2 plays a key role in neural development hence was the isoform of choice for this model. Behavioral abnormalities in this mouse model were robust in contrast to the *Ube3a* (2xTg) mouse developed by Smith et al. Although it showed normal growth in the first 2 weeks post birth and had normal motor and exploratory abilities, this model had fewer USV (Ultrasonic vocalization) at both early and later stages of development. Overexpression of Ube3a isoform 2 also led to anxiety and cognitive impairments. This mouse model exhibited a unique feature of reduced seizure threshold as well as volume reduction and dysmorphia of the hippocampus which was in accordance with the phenotype of Dup15 patients, where 60% patients have seizures and are known to have heteropias and dysplasias (Boronat et al., 2015).

## UBE3A and Autism: A Direct Link

Lossifov et al. conducted a whole genome sequencing study, where they found autism proband with a T485A missense mutation in *UBE3A* gene (Iossifov et al., 2014). Yi et al. also reported this mutation and suggested that it may lead to abnormally elevated activity of UBE3A (Yi et al., 2015). UBE3A maintains its optimum level via self-targeted degradation and is under tight control during normal brain development (Nuber et al., 1998; de Bie and Ciechanover, 2011; Mabb et al., 2011). T485A missense mutation disrupts the protein kinase A (PKA) phosphorylation site which is known to inhibit UBE3A ligase activity toward itself and its substrates. This leads to increase in UBE3A activity. Interestingly, this mutation also results in increased number of dendritic spine formation thus provides a mechanistic insight about how increased expression or activity of UBE3A could lead to altered synaptic function and plasticity. Scoles et al. found elevated level of UBE3A transcript and protein in post mortem brains of Dup15q individuals (Scoles et al., 2011). In accordance to this, Noor et al. have recently reported a family with 15q11.2 duplication limited to the *UBE3A* gene (Noor et al., 2015). The Comparative Genomic Hybridisation (aCGH) analysis revealed 129 kb duplication at chromosome 15q11.2 which encompassed the entire *UBE3A* gene with an overlap of all the three isoforms of UBE3A. They did not find any other clinically significant CNV. This patient was born to a 32 year old mother, via Cesarean section. The baby was diagnosed with infantile osteopetrosis at 9th day after birth for which she underwent bone marrow transplantation (BMT) at the age of 4 months. She suffered global developmental delay, although she could later communicate with little vocabulary. She is social with friends at school and also can make good eye contact. Family history of the patient revealed learning disability, anxiety, and depression on the patient's maternal side. These reports strongly indicate that UBE3A is one of the major contributors to the autistic phenotype in cases with maternal interstitial 15q11–13 duplication.

## Possible Therapies for Autism Targeting UBE3A: A Future Perspective

Realizing the potential of UBE3A in autism, Germain et al. in their iPSC lines from idic(15) patients restored normal UBE3A mRNA levels using antitumor antibiotic methamycin (Germain et al., 2014). However, this approach requires extensive study as methamycin affects the expression of genes other than *UBE3A*. Yi et al. reported that chronic treatment with drugs targeting PKA, like forskolin and rolipram, can turn down UBE3A activity in neurons. This raises the possibility of targeting upstream regulators of UBE3A in order to rescue autistic symptoms (Yi et al., 2015). Because the *UBE3A* is a paternally imprinted gene, its unsilencing could be a potential therapeutic strategy for AS and possibly other ASDs. Topoisomerase inhibitors have been found to unsilence the paternal *Ube3a* expression by inhibiting the expression of large noncoding antisense RNA transcript (UBE3A-ATS) (Huang et al., 2011). However, topoisomerase inhibitors could alter the expression of number of other genes and therefore could have severe detrimental effect that requires extensive study. Recently, antisense oligonucleotide of UBE3A-ATS has been shown to activate the paternal *Ube3a* and improve the behavioral abnormalities in AS mice (Meng et al., 2015). Similar strategy can also be tested to down-regulate Ube3a in rescuing autistic phenotypes in animals. Identification of novel substrates of UBE3A linked with synaptic function and plasticity could further lead to better understanding of the role of UBE3A in autism and AS that will eventually help in novel drug discovery. Detailed mechanistic study of neuronal circuit defects also need to be explored on models of ASD overexpressing UBE3A. Such studies will clearly elucidate the involvement of UBE3A in ASD and whether UBE3A alone could lead to such a robust autistic phenotype. Altogether, the finding of *UBE3A* as a candidate gene for autism is fairly new and holds promise in the future when mechanisms of action of UBE3A and its isoforms are elucidated, and upstream regulators of *UBE3A* gene are explored.

## Author Contributions

All authors listed have made a substantial, direct and intellectual contribution to the work, and approved it for publication.

### Conflict of Interest Statement

The authors declare that the research was conducted in the absence of any commercial or financial relationships that could be construed as a potential conflict of interest.

## References

[B1] Albrecht U. Sutcliffe J. S. Cattanach B. M. Beechey C. V. Armstrong D. Eichele G.. (1997). Imprinted expression of the murine Angelman syndrome gene, Ube3a, in hippocampal and Purkinje neurons. Nat. Genet. 17, 75–78. 10.1038/ng0997-75 9288101

[B2] Asperger H. (1991). Asperger and his syndrome, in Autism and Asperger Syndrome, ed Frith U. (New York, NY: Cambridge University Press), 37–92. 10.1017/CBO9780511526770.001

[B3] Berkel S. Marshall C. R. Weiss B. Howe J. Roeth R. Moog U.. (2010). Mutations in the SHANK2 synaptic scaffolding gene in autism spectrum disorder and mental retardation. Nat. Genet. 42, 489–491. 10.1038/ng.589 20473310

[B4] Boronat S. Mehan W. A. Shaaya E. A. Thibert R. L. Caruso P. (2015). Hippocampal abnormalities in magnetic resonance imaging (MRI) of 15q duplication syndromes. J. Child Neurol. 30, 333–338. 10.1177/0883073814538669 24985752

[B5] Chao H. T. Chen H. Samaco R. C. Xue M. Chahrour M. Yoo J.. (2010). Dysfunction in GABA signalling mediates autism-like stereotypies and Rett syndrome phenotypes. Nature 468, 263–269. 10.1038/nature09582 21068835 PMC3057962

[B6] Christensen J. Gronborg T. K. Sorensen M. J. Schendel D. Parner E. T. Pedersen L. H.. (2013). Prenatal valproate exposure and risk of autism spectrum disorders and childhood autism. JAMA 309, 1696–1703. 10.1001/jama.2013.2270 23613074 PMC4511955

[B7] Cook E. H. Jr Lindgren V. Leventhal B. L. Courchesne R. Lincoln A. (1997). Autism or atypical autism in maternally but not paternally derived proximal 15q duplication. Am. J. Hum. Genet. 60, 928–934. 9106540 PMC1712464

[B8] Copping N. A. S. Christian G. B. Ritter D. J. Islam M. S. Buscher N.. (2017). Neuronal overexpression of Ube3a isoform 2 causes behavioral impairments and neuroanatomical pathology relevant to 15q11.2-q13.3 duplication syndrome. Hum. Mol. Genet. 26, 3995–4010. 10.1093/hmg/ddx289 29016856 PMC5886211

[B9] de Bie P. Ciechanover A. (2011). Ubiquitination of E3 ligases: self-regulation of the ubiquitin system via proteolytic and non-proteolytic mechanisms. Cell Death Differ. 18, 1393–1402. 10.1038/cdd.2011.16 21372847 PMC3178436

[B10] de la Torre-Ubieta L. Won H. Stein J. L. Geschwind D. H. (2016). Advancing the understanding of autism disease mechanisms through genetics. Nat. Med. 22, 345–361. 10.1038/nm.4071 27050589 PMC5072455

[B11] Elegheert J. Kakegawa W. Clay J. E. Shanks N. F. Behiels E. Matsuda K.. (2016). Structural basis for integration of GluD receptors within synaptic organizer complexes. Science 353, 295–299. 10.1126/science.aae0104 27418511 PMC5291321

[B12] Ellegood J. Nakai N. Nakatani J. Henkelman M. Takumi T. Lerch J. (2015). Neuroanatomical phenotypes are consistent with autism-like behavioral phenotypes in the 15q11-13 duplication mouse model. Autism Res. 8, 545–555. 10.1002/aur.1469 25755142

[B13] Germain N. D. Chen P. F. Plocik A. M. Glatt-Deeley H. Brown J. Fink J. J.. (2014). Gene expression analysis of human induced pluripotent stem cell-derived neurons carrying copy number variants of chromosome 15q11-q13.1. Mol. Autism 5:44. 10.1186/2040-2392-5-44 25694803 PMC4332023

[B14] Gillberg C. (1998). Chromosomal disorders and autism. J. Autism Dev. Disord. 28, 415–425. 10.1023/A:1026004505764 9813777

[B15] Glessner J. T. Wang K. Cai G. Korvatska O. Kim C. E. Wood S.. (2009). Autism genome-wide copy number variation reveals ubiquitin and neuronal genes. Nature 459, 569–573. 10.1038/nature07953 19404257 PMC2925224

[B16] Godavarthi S. K. Dey P. Maheshwari M. Jana N. R. (2012). Defective glucocorticoid hormone receptor signaling leads to increased stress and anxiety in a mouse model of Angelman syndrome. Hum. Mol. Genet. 21, 1824–1834. 10.1093/hmg/ddr614 22215440

[B17] Greer P. L. Hanayama R. Bloodgood B. L. Mardinly A. R. Lipton D. M. Flavell S. W.. (2010). The Angelman Syndrome protein Ube3A regulates synapse development by ubiquitinating arc. Cell 140, 704–716. 10.1016/j.cell.2010.01.026 20211139 PMC2843143

[B18] Guan J. S. Haggarty S. J. Giacometti E. Dannenberg J. H. Joseph N. Gao J.. (2009). HDAC2 negatively regulates memory formation and synaptic plasticity. Nature 459, 55–60. 10.1038/nature07925 19424149 PMC3498958

[B19] Heck D. H. Zhao Y. Roy S. LeDoux M. S. Reiter L. T. (2008). Analysis of cerebellar function in Ube3a-deficient mice reveals novel genotype-specific behaviors. Hum. Mol. Genet. 17, 2181–2189. 10.1093/hmg/ddn117 18413322 PMC2902285

[B20] Hershko A. Ciechanover A. (1992). The ubiquitin system for protein degradation. Annu. Rev. Biochem. 61, 761–807. 10.1146/annurev.bi.61.070192.003553 1323239

[B21] Hogart A. Wu D. LaSalle J. M. Schanen N. C. (2010). The comorbidity of autism with the genomic disorders of chromosome 15q11.2-q13. Neurobiol. Dis. 38, 181–191. 10.1016/j.nbd.2008.08.011 18840528 PMC2884398

[B22] Huang H. S. Allen J. A. Mabb A. M. King I. F. Miriyala J. Taylor-Blake B.. (2011). Topoisomerase inhibitors unsilence the dormant allele of Ube3a in neurons. Nature 481, 185–189. 10.1038/nature10726 22190039 PMC3257422

[B23] Iijima T. Emi K. Yuzaki M. (2009). Activity-dependent repression of Cbln1 expression: mechanism for developmental and homeostatic regulation of synapses in the cerebellum. J. Neurosci. 29, 5425–5434. 10.1523/JNEUROSCI.4473-08.2009 19403810 PMC6665857

[B24] Iossifov I. O'Roak B. J. Sanders S. J. Ronemus M. Krumm N. Levy D.. (2014). The contribution of de novo coding mutations to autism spectrum disorder. Nature 515, 216–221. 10.1038/nature13908 25363768 PMC4313871

[B25] Jamal I. Kumar V. Vatsa N. Shekhar S. Singh B. K. Sharma A.. (2017). Rescue of altered HDAC activity recovers behavioural abnormalities in a mouse model of Angelman syndrome. Neurobiol. Dis. 105, 99–108. 10.1016/j.nbd.2017.05.010 28576709

[B26] Jiang Y. H. Armstrong D. Albrecht U. Atkins C. M. Noebels J. L. Eichele G.. (1998). Mutation of the Angelman ubiquitin ligase in mice causes increased cytoplasmic p53 and deficits of contextual learning and long-term potentiation. Neuron 21, 799–811. 10.1016/S0896-6273(00)80596-6 9808466

[B27] Kanner L. (1968). Autistic disturbances of affective contact. Acta Paedopsychiatr. 35, 100–136. 4880460

[B28] Kaphzan H. Buffington S. A. Jung J. I. Rasband M. N. Klann E. (2011). Alterations in intrinsic membrane properties and the axon initial segment in a mouse model of Angelman syndrome. J. Neurosci. 31, 17637–17648. 10.1523/JNEUROSCI.4162-11.2011 22131424 PMC3483031

[B29] Kaphzan H. Hernandez P. Jung J. I. Cowansage K. K. Deinhardt K. Chao M. V.. (2012). Reversal of impaired hippocampal long-term potentiation and contextual fear memory deficits in Angelman syndrome model mice by ErbB inhibitors. Biol. Psychiatry 72, 182–190. 10.1016/j.biopsych.2012.01.021 22381732 PMC3368039

[B30] Karimi P. Kamali E. Mousavi S. M. Karahmadi M. (2017). Environmental factors influencing the risk of autism. J. Res. Med. Sci. 22:27. 10.4103/1735-1995.200272 28413424 PMC5377970

[B31] Kashiwagi A. Meguro M. Hoshiya H. Haruta M. Ishino F. Shibahara T.. (2003). Predominant maternal expression of the mouse Atp10c in hippocampus and olfactory bulb. J. Hum. Genet. 48, 194–198. 10.1007/s10038-003-0009-3 12730723

[B32] Kayashima T. Yamasaki K. Joh K. Yamada T. Ohta T. Yoshiura K.. (2003). Atp10a, the mouse ortholog of the human imprinted ATP10A gene, escapes genomic imprinting. Genomics 81, 644–647. 10.1016/S0888-7543(03)00077-6 12782135

[B33] Khan O. Y. Fu G. Ismail A. Srinivasan S. Cao X. Tu Y.. (2006). Multifunction steroid receptor coactivator, E6-associated protein, is involved in development of the prostate gland. Mol. Endocrinol. 20, 544–559. 10.1210/me.2005-0110 16254014

[B34] Kim S. A. Kim J. H. Park M. Cho I. H. Yoo H. J. (2006). Association of GABRB3 polymorphisms with autism spectrum disorders in Korean trios. Neuropsychobiology 54, 160–165. 10.1159/000098651 17230033

[B35] Kishino T. Wagstaff J. (1998). Genomic organization of the UBE3A/E6-AP gene and related pseudogenes. Genomics 47, 101–107. 10.1006/geno.1997.5093 9465301

[B36] Klauck S. M. (2006). Genetics of autism spectrum disorder. Eur. J. Hum. Genet. 14, 714–720. 10.1038/sj.ejhg.5201610 16721407

[B37] Krishnan V. Stoppel D. C. Nong Y. Johnson M. A. Nadler M. J. Ozkaynak E.. (2017). Autism gene Ube3a and seizures impair sociability by repressing VTA Cbln1. Nature 543, 507–512. 10.1038/nature21678 28297715 PMC5364052

[B38] Kuhnle S. Mothes B. Matentzoglu K. Scheffner M. (2013). Role of the ubiquitin ligase E6AP/UBE3A in controlling levels of the synaptic protein Arc. Proc. Natl. Acad. Sci. U.S.A. 110, 8888–8893. 10.1073/pnas.1302792110 23671107 PMC3670309

[B39] LaSalle J. M. Reiter L. T. Chamberlain S. J. (2015). Epigenetic regulation of UBE3A and roles in human neurodevelopmental disorders. Epigenomics 7, 1213–1228. 10.2217/epi.15.70 26585570 PMC4709177

[B40] Lossie A. C. Whitney M. M. Amidon D. Dong H. J. Chen P. Theriaque D.. (2001). Distinct phenotypes distinguish the molecular classes of Angelman syndrome. J. Med. Genet. 38, 834–845. 10.1136/jmg.38.12.834 11748306 PMC1734773

[B41] Mabb A. M. Judson M. C. Zylka M. J. Philpot B. D. (2011). Angelman syndrome: insights into genomic imprinting and neurodevelopmental phenotypes. Trends Neurosci. 34, 293–303. 10.1016/j.tins.2011.04.001 21592595 PMC3116240

[B42] Malzac P. Webber H. Moncla A. Graham J. M. Kukolich M. Williams C.. (1998). Mutation analysis of UBE3A in Angelman syndrome patients. Am. J. Hum. Genet. 62, 1353–1360. 10.1086/301877 9585605 PMC1377156

[B43] Margolis S. S. Salogiannis J. Lipton D. M. Mandel-Brehm C. Wills Z. P. Mardinly A. R.. (2010). EphB-mediated degradation of the RhoA GEF Ephexin5 relieves a developmental brake on excitatory synapse formation. Cell 143, 442–455. 10.1016/j.cell.2010.09.038 21029865 PMC2967209

[B44] Matsuda K. Miura E. Miyazaki T. Kakegawa W. Emi K. Narumi S.. (2010). Cbln1 is a ligand for an orphan glutamate receptor delta2, a bidirectional synapse organizer. Science 328, 363–368. 10.1126/science.1185152 20395510

[B45] Matsuda K. Yuzaki M. (2011). Cbln family proteins promote synapse formation by regulating distinct neurexin signaling pathways in various brain regions. Eur. J. Neurosci. 33, 1447–1461. 10.1111/j.1460-9568.2011.07638.x 21410790

[B46] Meguro M. Kashiwagi A. Mitsuya K. Nakao M. Kondo I. Saitoh S.. (2001). A novel maternally expressed gene, ATP10C, encodes a putative aminophospholipid translocase associated with Angelman syndrome. Nat. Genet. 28, 19–20. 10.1038/ng0501-19 11326269

[B47] Meng L. Ward A. J. Chun S. Bennett C. F. Beaudet A. L. Rigo F. (2015). Towards a therapy for Angelman syndrome by targeting a long non-coding RNA. Nature 518, 409–412. 10.1038/nature13975 25470045 PMC4351819

[B48] Miao S. Chen R. Ye J. Tan G. H. Li S. Zhang J.. (2013). The Angelman syndrome protein Ube3a is required for polarized dendrite morphogenesis in pyramidal neurons. J. Neurosci. 33, 327–333. 10.1523/JNEUROSCI.2509-12.2013 23283345 PMC6618628

[B49] Mishra A. Dikshit P. Purkayastha S. Sharma J. Nukina N. Jana N. R. (2008). E6-AP promotes misfolded polyglutamine proteins for proteasomal degradation and suppresses polyglutamine protein aggregation and toxicity. J. Biol. Chem. 283, 7648–7656. 10.1074/jbc.M706620200 18201976

[B50] Mishra A. Godavarthi S. K. Jana N. R. (2009a). UBE3A/E6-AP regulates cell proliferation by promoting proteasomal degradation of p27. Neurobiol. Dis. 36, 26–34. 10.1016/j.nbd.2009.06.010 19591933

[B51] Mishra A. Godavarthi S. K. Maheshwari M. Goswami A. Jana N. R. (2009b). The ubiquitin ligase E6-AP is induced and recruited to aggresomes in response to proteasome inhibition and may be involved in the ubiquitination of Hsp70-bound misfolded proteins. J. Biol. Chem. 284, 10537–10545. 10.1074/jbc.M806804200 19233847 PMC2667740

[B52] Mishra A. Jana N. R. (2008). Regulation of turnover of tumor suppressor p53 and cell growth by E6-AP, a ubiquitin protein ligase mutated in Angelman mental retardation syndrome. Cell. Mol. Life Sci. 65, 656–666. 10.1007/s00018-007-7476-1 18193166 PMC11131694

[B53] Moncla A. Malzac P. Livet M. O. Voelckel M. A. Mancini J. Delaroziere J. C.. (1999). Angelman syndrome resulting from UBE3A mutations in 14 patients from eight families: clinical manifestations and genetic counselling. J. Med. Genet. 36, 554–560. 10424818 PMC1734398

[B54] Morrow E. M. Yoo S. Y. Flavell S. W. Kim T. K. Lin Y. Hill R. S.. (2008). Identifying autism loci and genes by tracing recent shared ancestry. Science 321, 218–223. 10.1126/science.1157657 18621663 PMC2586171

[B55] Mulherkar S. A. Sharma J. Jana N. R. (2009). The ubiquitin ligase E6-AP promotes degradation of alpha-synuclein. J. Neurochem. 110, 1955–1964. 10.1111/j.1471-4159.2009.06293.x 19645749

[B56] Nakatani J. Tamada K. Hatanaka F. Ise S. Ohta H. Inoue K.. (2009). Abnormal behavior in a chromosome-engineered mouse model for human 15q11-13 duplication seen in autism. Cell 137, 1235–1246. 10.1016/j.cell.2009.04.024 19563756 PMC3710970

[B57] Nawaz Z. Lonard D. M. Smith C. L. Lev-Lehman E. Tsai S. Y. Tsai M. J.. (1999). The Angelman syndrome-associated protein, E6-AP, is a coactivator for the nuclear hormone receptor superfamily. Mol. Cell. Biol. 19, 1182–1189. 10.1128/MCB.19.2.1182 9891052 PMC116047

[B58] Nishimura Y. Martin C. L. Vazquez-Lopez A. Spence S. J. Alvarez-Retuerto A. I. Sigman M.. (2007). Genome-wide expression profiling of lymphoblastoid cell lines distinguishes different forms of autism and reveals shared pathways. Hum. Mol. Genet. 16, 1682–1698. 10.1093/hmg/ddm116 17519220

[B59] Noor A. Dupuis L. Mittal K. Lionel A. C. Marshall C. R. Scherer S. W.. (2015). 15q11.2 duplication encompassing only the UBE3A gene is associated with developmental delay and neuropsychiatric phenotypes. Hum. Mutat. 36, 689–693. 10.1002/humu.22800 25884337

[B60] Nuber U. Schwarz S. E. Scheffner M. (1998). The ubiquitin-protein ligase E6-associated protein (E6-AP) serves as its own substrate. Eur. J. Biochem. 254, 643–649. 10.1046/j.1432-1327.1998.2540643.x 9688277

[B61] Nurmi E. L. Bradford Y. Chen Y. Hall J. Arnone B. Gardiner M. B.. (2001). Linkage disequilibrium at the Angelman syndrome gene UBE3A in autism families. Genomics 77, 105–113. 10.1006/geno.2001.6617 11543639

[B62] Oda H. Kumar S. Howley P. M. (1999). Regulation of the Src family tyrosine kinase Blk through E6AP-mediated ubiquitination. Proc. Natl. Acad. Sci. U.S.A. 96, 9557–9562. 10.1073/pnas.96.17.9557 10449731 PMC22247

[B63] Reiter L. T. Seagroves T. N. Bowers M. Bier E. (2006). Expression of the Rho-GEF Pbl/ECT2 is regulated by the UBE3A E3 ubiquitin ligase. Hum. Mol. Genet. 15, 2825–2835. 10.1093/hmg/ddl225 16905559 PMC3742451

[B64] Roberts E. M. English P. B. Grether J. K. Windham G. C. Somberg L. Wolff C. (2007). Maternal residence near agricultural pesticide applications and autism spectrum disorders among children in the California Central Valley. Environ. Health Perspect. 115, 1482–1489. 10.1289/ehp.10168 17938740 PMC2022638

[B65] Saitoh S. Buiting K. Cassidy S. B. Conroy J. M. Driscoll D. J. Gabriel J. M.. (1997). Clinical spectrum and molecular diagnosis of Angelman and Prader-Willi syndrome patients with an imprinting mutation. Am. J. Med. Genet. 68, 195–206. 10.1002/(SICI)1096-8628(19970120)68:2<195::AID-AJMG15>3.0.CO;2-P 9028458

[B66] Sato M. Stryker M. P. (2010). Genomic imprinting of experience-dependent cortical plasticity by the ubiquitin ligase gene Ube3a. Proc. Natl. Acad. Sci. U.S.A. 107, 5611–5616. 10.1073/pnas.1001281107 20212164 PMC2851788

[B67] Scheffner M. Huibregtse J. M. Vierstra R. D. Howley P. M. (1993). The HPV-16 E6 and E6-AP complex functions as a ubiquitin-protein ligase in the ubiquitination of p53. Cell 75, 495–505. 10.1016/0092-8674(93)90384-3 8221889

[B68] Schroer R. J. Phelan M. C. Michaelis R. C. Crawford E. C. Skinner S. A. Cuccaro M.. (1998). Autism and maternally derived aberrations of chromosome 15q. Am. J. Med. Genet. 76, 327–336. 10.1002/(SICI)1096-8628(19980401)76:4<327::AID-AJMG8>3.0.CO;2-M 9545097

[B69] Scoles H. A. Urraca N. Chadwick S. W. Reiter L. T. Lasalle J. M. (2011). Increased copy number for methylated maternal 15q duplications leads to changes in gene and protein expression in human cortical samples. Mol. Autism 2:19. 10.1186/2040-2392-2-19 22152151 PMC3287113

[B70] Sebat J. Lakshmi B. Malhotra D. Troge J. Lese-Martin C. Walsh T.. (2007). Strong association of de novo copy number mutations with autism. Science 316, 445–449. 10.1126/science.1138659 17363630 PMC2993504

[B71] Shao Y. Cuccaro M. L. Hauser E. R. Raiford K. L. Menold M. M. Wolpert C. M.. (2003). Fine mapping of autistic disorder to chromosome 15q11-q13 by use of phenotypic subtypes. Am. J. Hum. Genet. 72, 539–548. 10.1086/367846 12567325 PMC1180230

[B72] Shi S. Q. Bichell T. J. Ihrie R. A. Johnson C. H. (2015). Ube3a imprinting impairs circadian robustness in Angelman syndrome models. Curr. Biol. 25, 537–545. 10.1016/j.cub.2014.12.047 25660546 PMC4348236

[B73] Smith C. L. DeVera D. G. Lamb D. J. Nawaz Z. Jiang Y. H. Beaudet A. L.. (2002). Genetic ablation of the steroid receptor coactivator-ubiquitin ligase, E6-AP, results in tissue-selective steroid hormone resistance and defects in reproduction. Mol. Cell. Biol. 22, 525–535. 10.1128/MCB.22.2.525-535.2002 11756548 PMC139730

[B74] Smith S. E. Zhou Y. D. Zhang G. Jin Z. Stoppel D. C. Anderson M. P. (2011). Increased gene dosage of Ube3a results in autism traits and decreased glutamate synaptic transmission in mice. Sci. Transl. Med. 3:103ra97. 10.1126/scitranslmed.3002627 21974935 PMC3356696

[B75] Steffenburg S. Gillberg C. L. Steffenburg U. Kyllerman M. (1996). Autism in Angelman syndrome: a population-based study. Pediatr. Neurol. 14, 131–136. 10.1016/0887-8994(96)00011-2 8703225

[B76] Sun J. Zhu G. Liu Y. Standley S. Ji A. Tunuguntla R.. (2015). UBE3A regulates synaptic plasticity and learning and memory by controlling SK2 channel endocytosis. Cell Rep. 12, 449–461. 10.1016/j.celrep.2015.06.023 26166566 PMC4520703

[B77] Thomas J. A. Johnson J. Peterson Kraai T. L. Wilson R. Tartaglia N. LeRoux J.. (2003). Genetic and clinical characterization of patients with an interstitial duplication 15q11-q13, emphasizing behavioral phenotype and response to treatment. Am. J. Med. Genet. A 119A, 111–120. 10.1002/ajmg.a.10176 12749048

[B78] Urraca N. Cleary J. Brewer V. Pivnick E. K. McVicar K. Thibert R. L.. (2013). The interstitial duplication 15q11.2-q13 syndrome includes autism, mild facial anomalies and a characteristic EEG signature. Autism Res. 6, 268–279. 10.1002/aur.1284 23495136 PMC3884762

[B79] Valluy J. Bicker S. Aksoy-Aksel A. Lackinger M. Sumer S. Fiore R.. (2015). A coding-independent function of an alternative Ube3a transcript during neuronal development. Nat. Neurosci. 18, 666–673. 10.1038/nn.3996 25867122

[B80] Volk H. E. Lurmann F. Penfold B. Hertz-Picciotto I. McConnell R. (2013). Traffic-related air pollution, particulate matter, and autism. JAMA Psychiatry 70, 71–77. 10.1001/jamapsychiatry.2013.266 23404082 PMC4019010

[B81] Vorstman J. A. Staal W. G. van Daalen E. van Engeland H. Hochstenbach P. F. Franke L. (2006). Identification of novel autism candidate regions through analysis of reported cytogenetic abnormalities associated with autism. Mol. Psychiatry 11, 18–28. 10.1038/sj.mp.4001757 16205736

[B82] Vorstman J. A. S. Parr J. R. Moreno-De-Luca D. Anney R. J. L. Nurnberger J. I. Jr Hallmayer, J. F. (2017). Autism genetics: opportunities and challenges for clinical translation. Nat. Rev. Genet. 18, 362–376. 10.1038/nrg.2017.4 28260791

[B83] Wallace M. L. Burette A. C. Weinberg R. J. Philpot B. D. (2012). Maternal loss of Ube3a produces an excitatory/inhibitory imbalance through neuron type-specific synaptic defects. Neuron 74, 793–800. 10.1016/j.neuron.2012.03.036 22681684 PMC3372864

[B84] Weeber E. J. Jiang Y. H. Elgersma Y. Varga A. W. Carrasquillo Y. Brown S. E.. (2003). Derangements of hippocampal calcium/calmodulin-dependent protein kinase II in a mouse model for Angelman mental retardation syndrome. J. Neurosci. 23, 2634–2644. 10.1523/JNEUROSCI.23-07-02634.2003 12684449 PMC6742065

[B85] Wei P. Pattarini R. Rong Y. Guo H. Bansal P. K. Kusnoor S. V.. (2012). The Cbln family of proteins interact with multiple signaling pathways. J. Neurochem. 121, 717–729. 10.1111/j.1471-4159.2012.07648.x 22220752 PMC3342465

[B86] Weiss L. A. Shen Y. Korn J. M. Arking D. E. Miller D. T. Fossdal R.. (2008). Association between microdeletion and microduplication at 16p11.2 and autism. N. Engl. J. Med. 358, 667–675. 10.1056/NEJMoa075974 18184952

[B87] Williams C. A. Lossie A. Driscoll D. Unit R. C. P. (2001). Angelman syndrome: mimicking conditions and phenotypes. Am. J. Med. Genet. 101, 59–64. 10.1002/ajmg.1316 11343340

[B88] Yamamoto Y. Huibregtse J. M. Howley P. M. (1997). The human E6-AP gene (UBE3A) encodes three potential protein isoforms generated by differential splicing. Genomics 41, 263–266. 10.1006/geno.1997.4617 9143503

[B89] Yashiro K. Riday T. T. Condon K. H. Roberts A. C. Bernardo D. R. Prakash R.. (2009). Ube3a is required for experience-dependent maturation of the neocortex. Nat. Neurosci. 12, 777–783. 10.1038/nn.2327 19430469 PMC2741303

[B90] Yi J. J. Berrios J. Newbern J. M. Snider W. D. Philpot B. D. Hahn K. M.. (2015). An Autism-Linked Mutation Disables Phosphorylation Control of UBE3A. Cell 162, 795–807. 10.1016/j.cell.2015.06.045 26255772 PMC4537845

